# Beyond funding: Acknowledgement patterns in biomedical, natural and social sciences

**DOI:** 10.1371/journal.pone.0185578

**Published:** 2017-10-04

**Authors:** Adèle Paul-Hus, Adrián A. Díaz-Faes, Maxime Sainte-Marie, Nadine Desrochers, Rodrigo Costas, Vincent Larivière

**Affiliations:** 1 École de bibliothéconomie et des sciences de l'information, Université de Montréal Montreal, Quebec, Canada; 2 INGENIO (CSIC-UPV), Universitat Politècnica de València, Valencia, Spain; 3 Centre for Science and Technology Studies (CWTS), Leiden University, Leiden, the Netherlands; 4 Centre for Research on Evaluation, Science and Technology (CREST), Stellenbosch University, Stellenbosch, South Africa; 5 Centre Interuniversitaire de Recherche sur la Science et la Technologie (CIRST), Observatoire des Sciences et des Technologies (OST), Université du Québec à Montréal, Montreal, Quebec, Canada; Max Planck Institute, GERMANY

## Abstract

For the past 50 years, acknowledgments have been studied as important paratextual traces of research practices, collaboration, and infrastructure in science. Since 2008, funding acknowledgments have been indexed by Web of Science, supporting large-scale analyses of research funding. Applying advanced linguistic methods as well as Correspondence Analysis to more than one million acknowledgments from research articles and reviews published in 2015, this paper aims to go beyond funding disclosure and study the main types of contributions found in acknowledgments on a large scale and through disciplinary comparisons. Our analysis shows that technical support is more frequently acknowledged by scholars in Chemistry, Physics and Engineering. Earth and Space, Professional Fields, and Social Sciences are more likely to acknowledge contributions from colleagues, editors, and reviewers, while Biology acknowledgments put more emphasis on logistics and fieldwork-related tasks. Conflicts of interest disclosures (or lack of thereof) are more frequently found in acknowledgments from Clinical Medicine, Health and, to a lesser extent, Psychology. These results demonstrate that acknowledgment practices truly do vary across disciplines and that this can lead to important further research beyond the sole interest in funding.

## Introduction

For the past 50 years, acknowledgments have been studied as important paratextual traces of research practices, collaboration, infrastructure, and funding in science. In 1970, Crawford and Biderman [1] first used acknowledgments found in footnotes to analyse papers’ funding sources. Such analyses remained scarce for the following decades, mainly because of the lack of acknowledgment data on a large scale. This changed in 2008, when the Web of Science (WoS) started to index scientific papers’ acknowledgments, with a special focus on the funding information they contain. This has led to several analyses of trends in funding sources, as well as the relationship between funding, productivity, and the impact of publications (e.g., [2–6]). However, papers’ acknowledgments provide much more than funding information: they convey the indebtedness of authors to people and institutions whose contributions deserve to be publicly noted. As such, acknowledgments can be seen as expressions of scientific debt and have even been conceptualized as “super-citations” ([7]: p.106).

Although the concept of collaboration is typically measured through co-authorship, some of its dimensions can also be captured by examining the number and role of people acknowledged for their contributions [8]. Contributions and assistance acknowledged in scholarly papers have been described as a form of “subauthorship collaboration” ([9]: p.86). In that sense, acknowledgments help to reveal the—otherwise invisible—infrastructure that supports research. They show how colleagues, tools, equipment, materials, and grants are mobilized in the context of a research project [10]. Patel goes further in stating that “some illuminating details of group research are recorded nowhere else but in footnotes” ([9]: p.85). Investigating the forms of rewards associated with scientific collaboration, Laudel [11] found that certain types of scientific contributions are only rewarded through acknowledgments, while a significant portion of contributions remained completely invisible due to a lack of formal reward either in the byline or acknowledgment of a paper. This may be explained by both the lack of clear criteria for authorship and the fact that authors may not be familiar enough with such criteria [12].

Cronin [10] also put forward the value of acknowledgments, positioning them as data to be used alongside authorship and citation in the “reward triangle”, and thereby allowing “a more nuanced understanding of scholarly communication and interaction” ([13]: p.7). acknowledgments can be perceived as credit for contributions that complement authorship and could therefore be used to better understand collaboration and the division of labour in research. In short, acknowledgments have been studied as important paratextual [14] traces of collaboration, disciplinary research practices, and infrastructure in science for the past 50 years [15–16]; yet the lack of standardization in both their form and indexation, as well as an incomplete understanding of their function in the scholarly communication process, has led them to remain peripheral in the reward system of science.

In the 1990s, Cronin and McCain proposed a classification of acknowledgments by contribution types. The “peer interactive communication” (PIC) category was first described by McCain ([17]: p.512) and later adapted by Cronin [18] to refer to conceptual and cognitive contributions. Other categories included financial support, access to data and materials, technical assistance and manuscript preparation. In the following decades, acknowledgment analyses showed differences between disciplines in the prevalence of acknowledgments [19–20] and in the nature of support acknowledged [21].

Giles and Councill [22] was the first large-scale analysis of acknowledgments using natural language processing to extract named entities. Based on 188,052 acknowledgments automatically extracted from 335,000 research publications from the field of computer science, the authors proposed a measure of the impact of the most frequently acknowledged entities (AEs), the citation to acknowledgment (C/A) metric [22]. Giles and his colleagues pursued and presented this pioneering work in two conferences in 2012. First, Khabsa, Treeratpituk and Giles [23] extracted and disambiguated AEs from 526,930 acknowledgments from publications in computer science, mathematics, and statistics and found a correlation between the number of acknowledgments received by individuals and their h-index. Second, Khabsa, Koppman and Giles [24] furthered the analysis of AEs, looking at the social networks between named entities extracted from acknowledgments in computer science and mathematics. These studies were the first ones to use natural language processing on large datasets of acknowledgments. In the present study, the focus is moved from named entities to the tasks and contributions acknowledged in scientific paper and a multidisciplinary perspective is presented, using publications from biomedical, natural and social sciences, in order to compare acknowledgments practices in different fields.

Two analyses of PIC using WoS acknowledgment data have also been performed recently [2, 25]. Costas and van Leeuwen [2] operationalized PIC acknowledgments as the presence of terms relating to peer review and informal communication, such as ‘referee’, ‘comment’, ‘discussion’, ‘reading’ and ‘advice’. Using that definition, PIC acknowledgments were associated with a lower level of co-authorship. Costas and van Leeuwen [2] also found that papers that included funding acknowledgments presented a higher impact in terms of citations when compared to papers without funding acknowledgments. Focusing on papers published by Spanish researchers, Díaz-Faes and Bordons [25] compared acknowledgments patterns in four disciplines—cardiac and cardiovascular systems, economics, evolutionary biology, statistics and probability—and found that PIC acknowledgments were mostly associated with theoretical and social research.

The objective of this paper is to go beyond the funding disclosure inherent to the acknowledgments index in the WoS and study the main types of contributions found in acknowledgments across various disciplines and in the differences they exhibit, More specifically, this study aims at answering the following research questions:

- What types of contributions are acknowledged?
- How do the types of acknowledged contributions vary by discipline?

Given the well-known disciplinary difference in authorship practices [26–28], one might expect to observe similar disciplinary differences in the types of contributions acknowledged.

## Data and methods

Data for this study were retrieved from the Web of Science’s Science Citation Index Expanded (SCI-E) and Social Sciences Citation Index (SSCI), which both include funding acknowledgment data. WoS started to collect funding acknowledgment data in August 2008; however, until 2015, only articles and reviews covered in the SCI-E were included for funding acknowledgment. In 2015, WoS began to collect and index funding acknowledgment from articles and reviews covered in the SSCI [29].

These data are structured in three fields: the “Funding Text” (FT), “Funding Agency” (FO) and “Grant Number” (FG). FT is the full text of the acknowledgments and therefore contains funding information, but also all other types of support acknowledged by the authors. As noted above, however, acknowledgments are only collected and indexed by WoS if they include funding source information; they must also be written in English. In order to be able to compare acknowledgments patterns from the disciplines covered by the SCI-E (natural and biomedical sciences) to the ones covered by the SSCI (social sciences), 2015 was chosen as the target year for data collection and analysis.

Acknowledgments from 2015 articles and reviews (hereafter referred to as papers) indexed in the SCI-E and the SSCI were extracted. The corpus includes a total of 1,009,411 acknowledgments for as many papers. Discipline assignation was done using the National Science Foundation (NSF) field classification of journals which assigns only one discipline specialty to each journal, preventing the possible double counts of papers [30].

In order to identify and discriminate between the different types of contribution mentioned in the acknowledgments we collected, we performed a linguistic analysis focusing on noun phrase patterns. Broadly speaking, nouns phrases (or nominal phrases) are groups of words centered on a given noun and which, together with zero or more constituents of various syntactical categories (or adnouns), perform the same grammatical function as single nouns (for example, ‘financial support’, compared to simply ‘support’).

Many linguistic pre-processing stages were necessary to efficiently extract noun phrases from the 1,009,411 acknowledgments of our dataset. First, the acknowledgments retrieved from the FT field were segmented into words using the Penn TreeBank tokenizer [31–32] of the Natural Language ToolKit [33]. In order to identify common nouns present in the corpus, each tokenized acknowledgment was then morphologically and syntactically analyzed using the Stanford Log-Linear Part-of-Speech (POS) Tagger [34–35], based on the Penn TreeBank English POS tagset [31–32].

With respect to the identification and extraction of noun phrases, various implementation alternatives are available, since the characterization of noun phrases is still an active field of research in linguistics. Noun phrase chunking was implemented using a modified version of the grammatical ruleset designed by Kim et al. [36] for keyword extraction. This ruleset consists of two rules. The first rule aims to detect the nominal components of a noun phrase, from a single noun such as ‘funding’ to a sequence of nouns and/or adjectives ending with a noun, as in ‘internal funding program’ [37]. The objective of the second rule is to merge any pair of consecutive nominal components identified by the first rule and separated by any preposition or subordinating conjunction (of, for, with, in…), such as in ‘technical assistance in bacterial challenge experiment degeneration’ [38]. However, as formulated by Kim et al. [36], this second rule only allows for one such merging, which prevents the full identification of noun phrases such as ‘strategy for gene discovery in schizophrenia’ [39] or ‘coating of functionalized polysaccharide with embedded nanoparticles’ [40]. In order to properly extract such noun phrases, the second rule by Kim et al. [36] was modified in its application to our dataset in order to allow for multiple mergings of this sort. Following these linguistic processing stages, the noun phrase extraction procedure yielded initial totals of 408,608 distinct noun phrases and 5,646,656 occurrences.

A minimal transformation and standardization of the data was then performed by removing all noun phrases (NPs) containing only references to grant numbers. This was done by removing all NPs containing actual numbers and one of the following substrings: ‘no.’, ‘no’, ‘number’, ‘ref.’). NPs corresponding to any single letter of the alphabet were also removed, along with any punctuation sign at either extremity of the NP.

A frequency score was generated for each NP extracted, providing the number of occurrences for each NP in the dataset. A threshold of at least two occurrences was applied, meaning that all NPs appearing only once (*hapax legomena*) were removed. Fifteen NPs were also removed because they were deemed to have no meaning for the purpose of the analysis (i.e., ‘Auspex’, ‘and/or publication’, ‘Da’, ‘der’, ‘du’, ‘fur’, ‘herein’, ‘http’, ‘la’, ‘none’, ‘part’, ‘portion’, ‘section’, ‘van’ and ‘year’). Finally, eight NPs were merged in order to standardize distinct versions of the same NP (e.g., ‘field work’ and ‘fieldwork’, ‘lab’ and ‘laboratory’). As a result of these operations, the final dataset was reduced to 97,766 distinct NPs and a total of 4,875,216 occurrences (see Supporting Information for the frequency distribution of all NPs in the corpus).

For the purposes of the analysis, the dataset was partitioned by discipline and a Correspondence Analysis (CA) [41–43] was applied to these subsets following the procedure described in Díaz-Faes and Bordons [25] and using a MATLAB program [44]. This multivariate exploratory method allows for the displaying of the associations between rows and columns of a contingency table in a low-dimensional space in such a way that the underlying lexical patterns can be brought to the foreground. The closer the points (NPs, but also disciplines) are on the map, the more related they are. In other words, if two disciplines are very close in terms of projection (the direction from the origin), they have similar acknowledgment patterns. It should be noted that the proximity between NPs and disciplines is measured by χ2 distances, meaning that the weight assigned to each element of the matrix is inversely proportional to the row and column marginal totals. Since WoS only indexes acknowledgments that include funding information, NPs related to funding are expected to be very frequent, whereas NPs related to credit for peer interactive communication, intellectual indebtedness, or any other forms of contribution will have lower frequencies. These lower-frequencies NPs will be highlighted by the CA method, revealing the hidden infrastructure that supports scientific research.

To perform the CA, NPs were grouped using k-means clustering with cosine similarity. A threshold of 2,000 occurrences was established (i.e., NPs that appear at least 2,000 times in total were included). Thresholds of 2,500 and 3,000 occurrences were also tested, and the disciplines that strongly contribute to each factor were very similar for the various threshold values. The 2,000-occurrences threshold corresponds to the 214 most frequent NPs of the dataset, which account for 74% of the total NP occurrences for the whole corpus (see Supporting Information for the frequency table and plot of the 214 NPs included in the analysis). The resulting matrix of 214 NPs x 12 disciplines was then analyzed. As shown in Table 1, by retaining fives axes, 88% of the variance is explained. As factors obtained are uncorrelated, the explained variance for a NP or discipline on a particular plane is the sum of the contributions to the axes forming this plane, and this specific value is known as ‘quality of representation’ (QLR). For the five axes shown in Table 2, there is a good QLR for most NPs and disciplines (see Supporting Information for detailed QLR by NP and by discipline). In order to test the robustness of our results, an alternative analysis was performed using relative thresholds normalized by discipline, instead of an absolute threshold of 2,000 occurrences. The CA was rerun using the 100 most frequent NPs by discipline (i.e., 287 NPs in total). Using this alternative dataset, extremely similar clusters were obtained. However, the explained variance of the resulting axes were weaker, which led us to keep the original method of selecting NPs by threshold of occurrences.

**Table 1:** Explained and cumulative variance for each axis.

	Explained Variance (%)	Cumulative Variance (%)
Axis 1	36.56	36.56
Axis 2	19.00	55.56
Axis 3	15.79	71.35
Axis 4	10.48	81.83
Axis 5	6.20	88.03

**Table 2:** Relative contributions of the factor to the element for disciplines (expressed as a percentage).

Column	Discipline	Axis1	Axis2	Axis3	Axis4	Axis5
1	Biology	8.2%	12.9%	11.9%	**28.6%**	0.9%
2	Biomedical Research	**57.8%**	16.6%	24.4%	0.2%	0.8%
3	Chemistry	26.8%	**36.7%**	14.4%	1.8%	3.9%
4	Clinical Medicine	**55.8%**	10.0%	30.6%	0.0%	2.8%
5	Earth and Space	**30.4%**	17.6%	17.4%	15.7%	7.1%
6	Engineering and Technology	**58.1%**	10.4%	8.3%	1.8%	1.1%
7	Health	10.3%	**30.9%**	9.5%	1.7%	19.7%
8	Mathematics	12.9%	2.4%	6.5%	**63.2%**	11.1%
9	Physics	**39.4%**	26.0%	3.8%	0.3%	1.8%
10	Professional Fields	16.3%	**42.2%**	2.6%	9.8%	23.7%
11	Psychology	2.5%	**42.9%**	0.1%	4.7%	10.9%
12	Social Sciences	14.9%	**40.2%**	4.9%	3.5%	26.7%

Table 2 presents the percentage of the variance explained in each axis for the 12 disciplines. QLR is shown on a scale of 0–100 points and only disciplines and NPs with at least 40% of the information accounted for by a particular plane (QLR ≥ 40) were selected for the visualization. Such a threshold ensures that the lexical patterns observed are representative of the discipline. In the cases of Biomedical Research, Chemistry, Clinical Medicine, Engineering and Technology, Physics, Professional Fields and Social Sciences, the first plane (formed by Axes 1 and 2) explains approximately 60% of the variance, while for Earth and Space and Psychology, Axes 1 and 2 explain less than 50% of the variance. However, for Biology (which is the most dispersed discipline) and Mathematics, the variance is mostly explained in a residual plane (formed by Axes 3 and 4), suggesting a different lexical pattern than the one found in the first plane. In the case of Biology, none of the axes explain particularly well the acknowledgment patterns of the discipline, which could be explained by its heterogeneous distribution and by the specific types of assistance acknowledged in Biology.

## Results

Table 3 details the presence of acknowledgments in the 2015 papers indexed in WoS and which, it bears repeating, must contain funding information in order to be included. It shows that acknowledgments are not evenly distributed across disciplines. While the large majority of papers from the natural and medical sciences indexed in WoS contain funding acknowledgments—with a percentage above 80% for Biomedical Research and Chemistry—less than half of papers in the social sciences present acknowledgments with the required funding information.

**Table 3:** Number of papers indexed in WoS (all and with funding acknowledgments) and percentage of papers with funding acknowledgments, by discipline (2015).

Discipline	All Papers	Papers with Acknow.	% with Acknow.
Biomedical Research	189,066	158,067	83.6%
Chemistry	151,947	123,806	81.5%
Earth and Space	92,238	72,922	79.1%
Physics	124,556	95,676	76.8%
Biology	105,279	76,281	72.5%
Mathematics	49,997	35,390	70.8%
Engineering and Technology	241,124	165,590	68.7%
Clinical Medicine	389,311	218,367	56.1%
Health	37,309	18,703	50.1%
Psychology	31,286	15,085	48.2%
Social Sciences	50,420	16,972	33.7%
Professional Fields	41,015	12,552	30.6%
Total	1,503,548	1,009,411	67.1%

**Fig 1:**
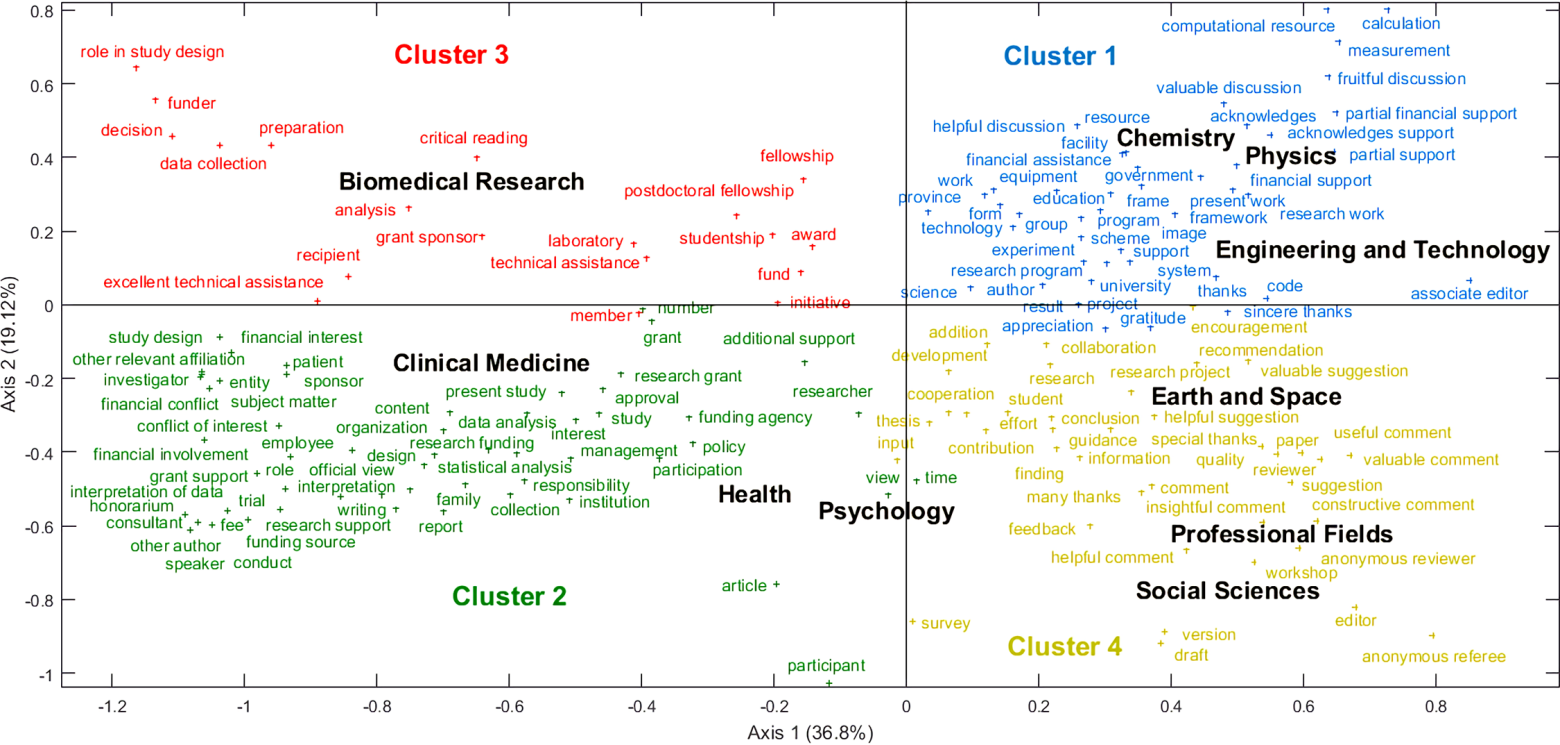
Bidimensional Correspondence Analysis for acknowledgments patterns by discipline (plane 1–2).

**Fig 2:**
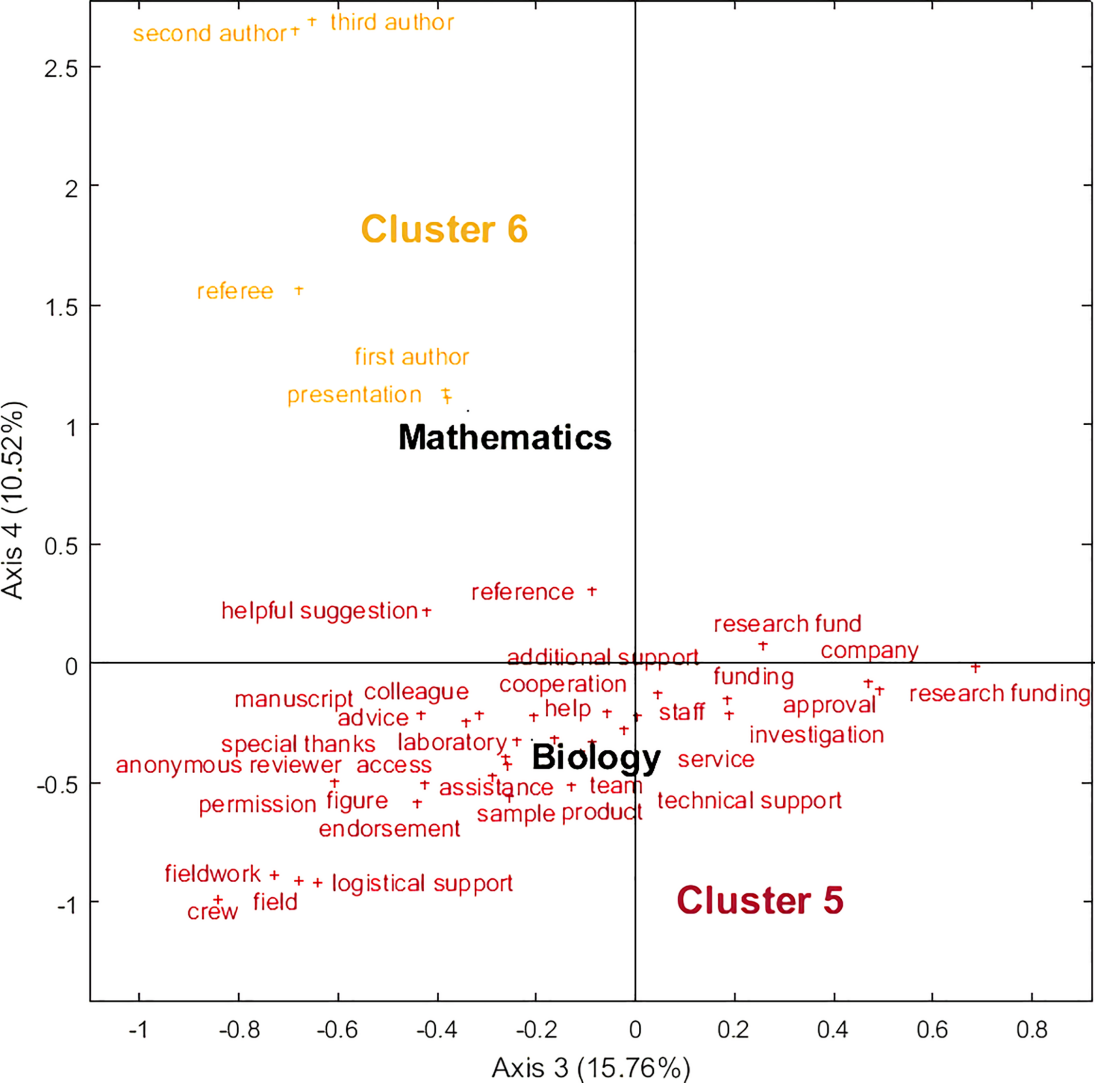
Bidimensional Correspondence Analysis for acknowledgments patterns by discipline (plane 3–4).

Figs 1 and 2 present the bidimensional CA of acknowledgment patterns by discipline. Six clusters can be distinguished. The first cluster, formed by Chemistry, Physics and Engineering and Technology, is marked by the acknowledgment of technical support with NPs referring to specific technical tasks (e.g., *image*, *equipment*, *computational resource*, *measurement*, *code*, *calculation*). NPs related to peer discussion (e.g., *fruitful discussion*, *helpful discussion*, *valuable discussion*) and to funding (e.g., *financial assistance*, *financial support*, *partial financial support*) also appear prominently in these natural sciences disciplines. Three main themes for these disciplines are therefore established as: technical tasks, PIC and funding.

In the second cluster, comprised of Clinical Medicine, Health and Psychology, NPs referring to funding and disclosure of potential conflict of interest (or lack of thereof) are dominant (e.g., *fee*, *honorarium*, *conflict of interest*, *consultant*, *employee*, *financial conflict*, *financial interest*, *financial involvement*, *sponsor*, *other relevant affiliation*). NPs specifically related to the experimental work involving the participation of human subjects also appear in the acknowledgment patterns of that cluster (e.g., *family*, *patient*, *participant*, *participation*, *trial*). Here, the main themes are funding-related ethics, and human subjects, which define the fields.

Interestingly, NPs associated with Psychology, a discipline often difficult to categorize, are located halfway between the applied health cluster (cluster 2) and the Social Sciences and Professional Fields (see cluster 4).

The third cluster consists of Biomedical Research and is mainly characterized by NPs referring to funding (e.g., *funder*, *grant sponsor*, *fund*, *fellowship*, *studentship*, *recipient*). There is also a prevalence of terms related to performing the research (e.g., *analysis*, *data collection*, *preparation*, *technical assistance*, *excellent technical assistance*). This cluster therefore sits at the juncture of finance and analysis.

Earth and Space, Professional Fields and Social Sciences are all in the fourth cluster. It is the cluster where peer communication and intellectual debt are clearly the dominant types of contributions acknowledged, with numerous NPs referring to input by reviewers, editors, and colleagues (e.g., *guidance*, *feedback*, *valuable suggestion*, *useful comment*, *helpful comment*, *valuable comment*, *insightful comment*, *editor*, *reviewer*, *anonymous reviewer*, *anonymous referee*). The presence of Earth and Space in that cluster illustrates its more heterogeneous profile in terms of acknowledgments, with information scattered on different axes, in a manner similar to what is found in Biology (see Table 3). This PIC- and peer-review-centric cluster is thus one that reaches across very different fields.

The fifth cluster consists of Biology (Fig 2) and is characterized by NPs related to the specific nature of experimental work in that field (e.g., *field*, *fieldwork*, *access*, *sample*, *laboratory*, *logistical support*, *technical support*). Furthermore, collaboration appears as an important aspect of acknowledgments in Biology with NPs such as *cooperation*, *crew*, *team*, and *staff*. Finally, Mathematics is found as a peripheral cluster (cluster 6) and is mainly characterized with NPs referring to authorship (e.g., *first author*, *second author*, *third author*) and the presence of the NP ‘referee’. These two fields hence stand alone, but Biology shows a potential for more specific comparisons with Clusters 1 and 3 in terms of technical work or analysis, and clusters 1 and 4 in terms of collaboration-related terms. Mathematics is the eccentric field, but shows the importance of collaboration, assigning individual responsibilities or funding to specific authors.

While we could have expected that NPs referring to the peer review process would be scattered across all disciplines (since peer review is a common denominator in all fields), acknowledgments directly thanking referees, reviewers and editors appear as a distinctive feature of the disciplines in the fourth cluster (Earth and Space, Professional Fields and Social Sciences) and in Mathematics, which stands alone as cluster 6. In the same way, NPs referring to funding take a different form in the second cluster—which regroups applied health disciplines (Clinical Medicine, Health and Psychology)—where ethical considerations and disclosure of conflict of interest appear as a dominant trend, while they did not appear as prevalent in the other clusters. In this language-based analysis, form and wording appear to have a capital influence. Indeed, the absence of given themes or topics in a cluster does not mean that said topics and themes are absent from a discipline altogether; it does, however, mean that the patterns formed by the NPs included in the analysis favour other, stronger trends in terms of disciplinary practices. Furthermore, variations in the wording of similar themes across disciplines, such as funding and PIC, could certainly be analysed further to see where the parallels between disciplinary cultures begin and end, as well as the weight of formulaic or required statements.

## Discussion and conclusion

As one might expect from WoS acknowledgment indexation—which, again, only includes acknowledgments if funding information is provided and if they are written in English—our results show that the proportion of papers with acknowledgments varies across disciplines, with a higher proportion of papers containing funding acknowledgments in the biomedical sciences (>80%), followed by the natural sciences (from 70% to 80%), clinical medicine (≅50%) and the social sciences (>30%). These results are in line with previous findings [2, 25, 29, 45].

As often discussed in the literature, acknowledgments found in scholarly papers provide a window on the otherwise invisible contributions made to research by individuals and organisations. Previous research has shown that these contributions—which are not considered sufficient to grant authorship, key to the accumulation of capital in modern science [10, 46]—have traditionally been grouped into similar types of categories since McCain [17] and Cronin et al. [18] proposed their taxonomies: conceptual and cognitive, financial support, access to data and materials, technical assistance, and manuscript preparation. A large body of research on acknowledgments has been published since these foundational models were proposed (see [16], for a meta-synthesis of the literature), and while there exists a sizeable amount of large-scale analyses of authorship and collaboration, their evolution over time, and the variations they exhibit across disciplines (e.g. [47–50]), no multidisciplinary large-scale study had previously analysed how acknowledged contributions vary across the fields of biomedical, natural and social sciences. Our quantitative, data-driven analysis offers a large-scale overview of the main disciplinary patterns found in terms of types of contributions acknowledged; this provides insight on the tasks and work valued in each field, the practices in acknowledging them, the trends they create with regard to how things are expressed, the importance given to certain aspects of research such as the peer review process or ethics-related statements, as well as on the division of labor, in certain cases. Our results are of particular interest in the case of the social sciences, since previous WoS-based studies, including our own [51], were mainly limited to SCI-E, as funding acknowledgment data for SSCI have only been collected since 2015. By applying advanced linguistic methods as well as Correspondence Analysis to 1,009,411 acknowledgments from papers published in 2015, this study contributes to provide a better understanding of acknowledgment practices by reaffirming but also expanding the main types of contributions found in the traditional taxonomies of acknowledgments proposed in 1990s [17, 18]. More importantly, our analysis highlights important disciplinary variations in the practices, trends and etiquette of acknowledging, all of which are direct reflections of different disciplinary cultures in research, collaboration, and scholarly communication itself.

Technical support was more frequently acknowledged by scholars in the natural sciences (Chemistry, Physics and Engineering). Earth and Space, Professional Fields, and Social Sciences were more likely to acknowledge contributions from colleagues, editors, and reviewers, and Biology acknowledgments put more emphasis on logistics and other fieldwork-related tasks. While Biomedical Research mostly acknowledged funding—which might be a reflection of the larger spectrum of contributions that lead to authorship in that specific discipline, and thus to funding disclosures from more authors [27]—Clinical Medicine, Health and, to a lesser extent, Psychology, were much more likely to include statements related to various forms of conflicts of interest. Conflicts of interest constitute important issues in clinical studies, given the potential consequences of fraud and unethical behaviour in these fields.

This suggests that acknowledgments are not confined to credit attribution, such as the traditional acknowledgment taxonomies seem to indicate [17, 18]. In fact, disclosures of potential conflicts of interest show the presence of pre-formulated statements recommended by funding bodies, ethical boards, or editorial requirements may influence language-based analyses of acknowledgments and reveal important disciplinary requirements and practices. Expressions associated with conflict of interest—or lack thereof—were mostly found in the clinical and applied health fields, in which disclosure of potential conflicts is made mandatory by most journals’ guidelines, as established by the largest consortium of medical journal editors, the International Committee of Medical Journal Editors [52]. These (self-)imposed statements obviously protect third parties, including the journals themselves, as well as the researchers. Interestingly, these traces of accountability add to the use of acknowledgments alongside authorship lists to attribute—or remove—responsibility in terms of the results published.

This leads to the importance of interpreting NPs in light of their original context, which the preliminary findings from ongoing qualitative analyses already show. These reveal interesting aspects to these NPs: keeping the example of conflict of interest disclosure, the NP ‘role in study design’ may not be the reflection of a task performed, but rather an indication that it was not, as in the sentence, “The funders had no role in study design, data collection and analysis, decision to publish, or preparation of the manuscript” [53]. Indeed, that NP is often associated with conflict of interest disclosure statements recommended by PLOS journals, PeerJ, and other scholarly communication venues. The NP ‘speaker’ is also linked to conflict of interest issues in Clinical Medicine, as in this typical example: “JMH has acted as a consultant, received grants, and acted as a speaker in activities sponsored by Astra-Zeneca, Eli Lilly and Company, Glaxo-SmithKline, and Lundbeck” [54]; here, the ‘speaker’ is the researcher and the use of the NP is related to disclosure. However, ‘speaker’ can also be used to describe a person other than the researcher in order to acknowledge their contribution, as in this example: “We would like to thank the following student research assistants: […] native speaker of English Stewart Campbell for proofreading the manuscript” [55]; in such cases, the use of the NP shifts back to collaboration and support. The qualitative coding of NPs in the context of their use constitutes the next phase of the project and will complement the findings presented here by adding various layers to our understanding of scientific acknowledgments. As this qualitative analysis calls for a reporting style based on thick description, it is best suited for another paper due to the time and space it requires, but it constitutes further research worth pursuing, as it will allow us to measure the relative weight of funding-related and non-funding-related uses of the NPs found in acknowledgments. This will in turn provide us with a better understanding of how acknowledgments indexed in WoS can support the analysis of scientific practices beyond the core concern with funding.

Papers’ acknowledgments provide much more than funding information. They shed light on otherwise invisible contributions that complement authorship and provide insight on researchers’ collaboration patterns, division of labour, and credit attribution practices. Furthermore, acknowledgment structures, lexicons, and uses do vary by discipline, and so this paratext can reveal much about researchers’ practices, the context in which they conduct research, and the specific aspects of the scholarly work they value or deem worth mentioning to protect themselves or those who support them. In many instances, acknowledgments remain the only space where such revealing details about specific and disciplinary research contexts are recorded. More forays into the role played by acknowledgments as testimonies to these practices, beyond funding, are needed to better understand how they can be used to illustrate disciplinary issues, differences, and similarities.

## Supporting information

S1 Fig Frequency distribution of noun phrases found in acknowledgments. (TIF)

S1 Table Frequency of the 214 most frequent noun phrases, by discipline. (DOCX)

S2 Table Quality of representation of the rows (cumulative contribution for each NP). (DOCX)

S3 Table Quality of representation of the columns (cumulative contribution for each discipline). (DOCX)
