# Supplementary figures and images for: Beyond funding: Acknowledgement patterns in biomedical, natural and social sciences
Source: PLoS One. 2017 Oct 4;12(10):e0185578. doi: 10.1371/journal.pone.0185578 (PMC5627922; doi:10.1371/journal.pone.0185578)

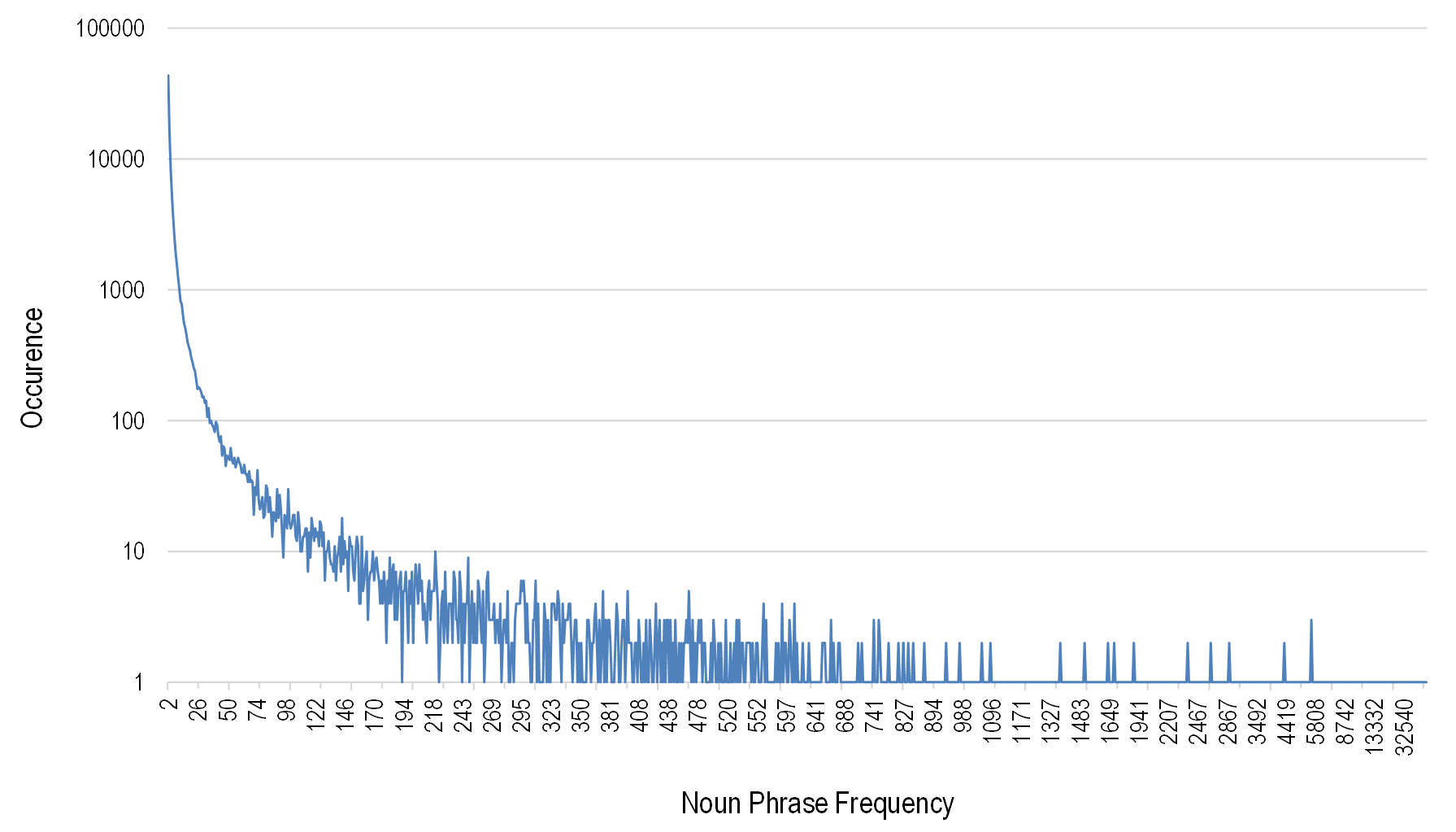

Supplement: S1 Fig — (TIF) [file pone.0185578.s001.tif]
