# Supplementary material for: Beyond funding: Acknowledgement patterns in biomedical, natural and social sciences
Source: PLoS One. 2017 Oct 4;12(10):e0185578. doi: 10.1371/journal.pone.0185578 (PMC5627922; doi:10.1371/journal.pone.0185578)
Supplement: S1 Table — (DOCX) [file pone.0185578.s002.docx]

S1 Table. Frequency of the 214 most frequent noun phrases, by discipline

| **Rank** | **Noun Phrases** | **Biology** | **Biomedical Research** | **Chemistry** | **Clinical Medicine** | **Earth and Space** | **Engineering and Technology** | **Health** | **Mathematics** | **Physics** | **Professional Fields** | **Psychology** | **Social Sciences** | **Total** |
| --- | --- | --- | --- | --- | --- | --- | --- | --- | --- | --- | --- | --- | --- | --- |
| 1 | work | 27038 | 83705 | 57640 | 79759 | 32782 | 94048 | 4609 | 14445 | 62636 | 3181 | 4202 | 3644 | 467689 |
| 2 | author | 23900 | 35134 | 39828 | 58117 | 27356 | 63986 | 7951 | 13460 | 26270 | 6351 | 4858 | 7308 | 314519 |
| 3 | grant | 14399 | 52953 | 12223 | 69030 | 16819 | 16372 | 3837 | 8079 | 9172 | 1756 | 4648 | 2123 | 211411 |
| 4 | study | 19381 | 32009 | 7624 | 73724 | 16214 | 10583 | 8271 | 470 | 3790 | 1962 | 4350 | 1992 | 180370 |
| 5 | research | 17981 | 20857 | 14714 | 25729 | 19783 | 28703 | 5506 | 9724 | 12642 | 5195 | 6791 | 7244 | 174869 |
| 6 | project | 17342 | 19255 | 17017 | 19069 | 19593 | 28145 | 2799 | 4858 | 14901 | 2243 | 1936 | 3520 | 150678 |
| 7 | financial support | 10617 | 10738 | 38990 | 11388 | 9460 | 31349 | 2119 | 1929 | 13712 | 2441 | 1037 | 4212 | 137992 |
| 8 | support | 8256 | 14795 | 15017 | 14852 | 17204 | 21579 | 2078 | 3488 | 16727 | 1768 | 1376 | 2711 | 119851 |
| 9 | manuscript | 16271 | 36332 | 2727 | 18837 | 15135 | 4266 | 1548 | 1647 | 3064 | 654 | 2013 | 1022 | 103516 |
| 10 | paper | 3895 | 2889 | 1355 | 5658 | 12175 | 13837 | 1214 | 4601 | 3795 | 2622 | 849 | 4446 | 57336 |
| 11 | program | 3947 | 7625 | 7036 | 6155 | 6699 | 8806 | 760 | 1112 | 6676 | 379 | 422 | 677 | 50294 |
| 12 | analysis | 2119 | 25144 | 2380 | 10872 | 2788 | 1751 | 1439 | 669 | 606 | 230 | 630 | 435 | 49063 |
| 13 | funding | 4448 | 7499 | 4758 | 8614 | 4203 | 4958 | 1271 | 460 | 2734 | 506 | 590 | 946 | 40987 |
| 14 | preparation | 1018 | 23015 | 673 | 6575 | 809 | 599 | 536 | 797 | 387 | 137 | 622 | 134 | 35302 |
| 15 | assistance | 6159 | 5438 | 3774 | 5829 | 4985 | 3711 | 583 | 75 | 1559 | 320 | 744 | 638 | 33815 |
| 16 | anonymous reviewer | 7538 | 1720 | 331 | 679 | 13006 | 3838 | 294 | 670 | 765 | 1382 | 608 | 2261 | 33092 |
| 17 | data collection | 1067 | 22639 | 307 | 5760 | 444 | 189 | 719 | 590 | 64 | 179 | 706 | 195 | 32859 |
| 18 | help | 5872 | 5516 | 3793 | 3914 | 5243 | 4147 | 258 | 290 | 2212 | 273 | 756 | 497 | 32771 |
| 19 | data | 2399 | 3657 | 1022 | 6986 | 11931 | 1926 | 1030 | 192 | 843 | 553 | 915 | 1086 | 32540 |
| 20 | decision | 500 | 22613 | 150 | 7125 | 210 | 201 | 616 | 617 | 69 | 63 | 319 | 56 | 32539 |
| 21 | thanks | 4728 | 2515 | 6049 | 2125 | 4906 | 3871 | 370 | 640 | 3034 | 461 | 413 | 1057 | 30169 |
| 22 | article | 1316 | 3497 | 632 | 8433 | 1720 | 1772 | 2976 | 741 | 474 | 1780 | 1845 | 3583 | 28769 |
| 23 | funder | 470 | 21115 | 75 | 4264 | 111 | 60 | 632 | 590 | 10 | 70 | 163 | 66 | 27626 |
| 24 | role in study design | 355 | 19967 | 54 | 3275 | 61 | 44 | 220 | 579 | 6 | 17 | 115 | 9 | 24702 |
| 25 | comment | 4003 | 2741 | 426 | 1583 | 5672 | 1818 | 290 | 1000 | 1533 | 862 | 532 | 1979 | 22439 |
| 26 | fellowship | 2237 | 5690 | 4355 | 4052 | 1115 | 1496 | 157 | 315 | 1256 | 41 | 219 | 171 | 21104 |
| 27 | fund | 2142 | 4250 | 2086 | 4700 | 1438 | 2299 | 330 | 387 | 1124 | 160 | 234 | 205 | 19355 |
| 28 | suggestion | 2234 | 1456 | 609 | 828 | 4866 | 2615 | 174 | 1921 | 1256 | 1004 | 250 | 1743 | 18956 |
| 29 | contract | 816 | 2228 | 1777 | 2581 | 4756 | 2972 | 264 | 405 | 1951 | 73 | 100 | 107 | 18030 |
| 30 | number | 1358 | 4306 | 1149 | 6272 | 886 | 1882 | 308 | 327 | 787 | 171 | 232 | 281 | 17959 |
| 31 | publication | 1075 | 1963 | 537 | 4050 | 1617 | 5018 | 711 | 141 | 1062 | 164 | 283 | 199 | 16820 |
| 32 | helpful discussion | 702 | 3282 | 2770 | 1390 | 1766 | 1691 | 26 | 494 | 3338 | 77 | 112 | 168 | 15816 |
| 33 | research grant | 1105 | 2660 | 992 | 6892 | 802 | 1288 | 331 | 311 | 588 | 247 | 327 | 256 | 15799 |
| 34 | technical assistance | 2687 | 4706 | 1355 | 3981 | 820 | 1001 | 52 | 10 | 594 | 14 | 161 | 43 | 15424 |
| 35 | member | 1387 | 4379 | 626 | 3949 | 1079 | 1078 | 390 | 242 | 719 | 201 | 362 | 275 | 14687 |
| 36 | contribution | 1690 | 1711 | 838 | 2913 | 3277 | 1694 | 556 | 108 | 748 | 257 | 431 | 348 | 14571 |
| 37 | discussion | 956 | 2639 | 1076 | 908 | 2754 | 1366 | 45 | 435 | 3670 | 135 | 117 | 423 | 14524 |
| 38 | helpful comment | 2272 | 1399 | 316 | 988 | 2921 | 1087 | 231 | 924 | 810 | 991 | 546 | 1875 | 14360 |
| 39 | staff | 3225 | 2004 | 553 | 3211 | 2102 | 813 | 523 | 36 | 674 | 135 | 375 | 304 | 13955 |
| 40 | material | 1382 | 1868 | 1389 | 2694 | 1174 | 2765 | 274 | 295 | 1118 | 243 | 216 | 328 | 13746 |
| 41 | view | 784 | 1312 | 337 | 3528 | 1278 | 1929 | 1101 | 171 | 706 | 731 | 601 | 1050 | 13528 |
| 42 | conflict of interest | 834 | 3081 | 111 | 7995 | 139 | 92 | 646 | 11 | 25 | 39 | 327 | 32 | 13332 |
| 43 | content | 378 | 3035 | 307 | 5801 | 530 | 513 | 1113 | 177 | 151 | 234 | 586 | 219 | 13044 |
| 44 | scholarship | 2529 | 2257 | 1812 | 2197 | 987 | 1621 | 209 | 172 | 575 | 61 | 194 | 147 | 12761 |
| 45 | laboratory | 2118 | 4735 | 833 | 2420 | 1087 | 771 | 39 | 26 | 370 | 25 | 127 | 51 | 12602 |
| 46 | facility | 1322 | 1953 | 2785 | 1073 | 1547 | 2081 | 46 | 52 | 1504 | 30 | 51 | 65 | 12509 |
| 47 | experiment | 1904 | 2089 | 1722 | 1580 | 889 | 2044 | 30 | 29 | 1507 | 65 | 356 | 147 | 12362 |
| 48 | use | 999 | 1598 | 1826 | 1010 | 3619 | 1755 | 83 | 58 | 1086 | 69 | 86 | 111 | 12300 |
| 49 | result | 599 | 1337 | 1128 | 2011 | 2027 | 2168 | 242 | 421 | 1295 | 158 | 146 | 337 | 11869 |
| 50 | first author | 1490 | 431 | 181 | 810 | 1292 | 1964 | 299 | 3324 | 396 | 454 | 727 | 310 | 11678 |
| 51 | award | 774 | 2548 | 1875 | 2712 | 1078 | 1115 | 228 | 216 | 607 | 63 | 213 | 108 | 11537 |
| 52 | sponsor | 482 | 3271 | 46 | 6568 | 252 | 398 | 163 | 9 | 56 | 30 | 79 | 49 | 11403 |
| 53 | access | 2509 | 1523 | 1395 | 719 | 2264 | 1099 | 126 | 42 | 666 | 114 | 103 | 328 | 10888 |
| 54 | framework | 784 | 988 | 1479 | 779 | 1376 | 2602 | 54 | 279 | 1758 | 93 | 60 | 214 | 10466 |
| 55 | review | 659 | 960 | 209 | 2734 | 1071 | 3160 | 270 | 22 | 1103 | 48 | 116 | 61 | 10413 |
| 56 | valuable comment | 1898 | 705 | 269 | 468 | 2124 | 1731 | 96 | 992 | 728 | 475 | 178 | 671 | 10335 |
| 57 | technical support | 1415 | 2091 | 1187 | 1883 | 1003 | 1547 | 62 | 25 | 879 | 23 | 111 | 59 | 10285 |
| 58 | anonymous referee | 1128 | 235 | 75 | 84 | 2453 | 954 | 81 | 1663 | 554 | 883 | 149 | 1964 | 10223 |
| 59 | editor | 1128 | 352 | 99 | 281 | 2600 | 1355 | 166 | 844 | 233 | 866 | 162 | 1414 | 9500 |
| 60 | constructive comment | 1423 | 513 | 141 | 278 | 4071 | 1137 | 55 | 451 | 301 | 342 | 110 | 472 | 9294 |
| 61 | reviewer | 1089 | 486 | 434 | 447 | 2791 | 1803 | 128 | 632 | 425 | 370 | 195 | 483 | 9283 |
| 62 | education | 745 | 1188 | 1598 | 1144 | 775 | 1977 | 75 | 353 | 920 | 100 | 54 | 79 | 9008 |
| 63 | collection | 1338 | 1178 | 191 | 4459 | 572 | 110 | 473 | 13 | 25 | 75 | 305 | 155 | 8894 |
| 64 | responsibility | 168 | 1448 | 291 | 3968 | 298 | 476 | 866 | 93 | 161 | 234 | 510 | 366 | 8879 |
| 65 | useful discussion | 196 | 712 | 892 | 207 | 1555 | 875 | 6 | 282 | 4012 | 21 | 20 | 58 | 8836 |
| 66 | research project | 1026 | 763 | 666 | 1149 | 1064 | 1857 | 222 | 286 | 537 | 402 | 190 | 580 | 8742 |
| 67 | special thanks | 1833 | 842 | 560 | 909 | 1791 | 1207 | 198 | 107 | 374 | 226 | 248 | 404 | 8699 |
| 68 | measurement | 298 | 554 | 3000 | 332 | 817 | 2018 | 5 | 1 | 1216 | 1 | 8 | 17 | 8267 |
| 69 | version | 1942 | 579 | 50 | 585 | 1633 | 425 | 221 | 355 | 162 | 594 | 471 | 1170 | 8187 |
| 70 | recipient | 561 | 3113 | 383 | 3380 | 198 | 178 | 84 | 27 | 77 | 17 | 56 | 19 | 8093 |
| 71 | grant sponsor | 206 | 2846 | 1186 | 2867 | 192 | 105 | 1 | 247 | 26 | 39 | 149 | 126 | 7990 |
| 72 | gratitude | 912 | 627 | 846 | 978 | 1019 | 1704 | 183 | 337 | 568 | 172 | 138 | 259 | 7743 |
| 73 | advice | 1376 | 1785 | 408 | 1118 | 1061 | 630 | 108 | 124 | 386 | 128 | 143 | 294 | 7561 |
| 74 | role | 152 | 1674 | 50 | 4287 | 186 | 60 | 788 | 15 | 24 | 52 | 214 | 56 | 7558 |
| 75 | writing | 260 | 1210 | 81 | 4171 | 210 | 121 | 747 | 60 | 72 | 91 | 271 | 163 | 7457 |
| 76 | participant | 240 | 686 | 33 | 2147 | 451 | 226 | 699 | 78 | 143 | 763 | 545 | 1368 | 7379 |
| 77 | official view | 113 | 1288 | 208 | 3311 | 188 | 184 | 741 | 65 | 108 | 100 | 454 | 113 | 6873 |
| 78 | fruitful discussion | 235 | 678 | 1084 | 218 | 812 | 1093 | 4 | 200 | 2374 | 10 | 27 | 34 | 6769 |
| 79 | opinion | 404 | 580 | 274 | 1077 | 717 | 1455 | 373 | 111 | 556 | 476 | 299 | 443 | 6765 |
| 80 | study design | 132 | 2605 | 32 | 3119 | 77 | 30 | 329 | 5 | 2 | 40 | 190 | 38 | 6599 |
| 81 | design | 247 | 1026 | 136 | 3369 | 192 | 308 | 719 | 12 | 178 | 61 | 187 | 45 | 6480 |
| 82 | foundation | 398 | 1248 | 674 | 1690 | 460 | 737 | 102 | 191 | 634 | 79 | 90 | 120 | 6423 |
| 83 | sample | 1172 | 1331 | 708 | 713 | 1218 | 639 | 11 | 2 | 506 | 7 | 31 | 50 | 6388 |
| 84 | collaboration | 722 | 709 | 386 | 1004 | 1023 | 859 | 180 | 107 | 768 | 112 | 118 | 181 | 6169 |
| 85 | resource | 204 | 774 | 1146 | 793 | 789 | 876 | 75 | 70 | 1260 | 22 | 55 | 31 | 6095 |
| 86 | development | 524 | 714 | 456 | 1394 | 963 | 885 | 237 | 64 | 421 | 146 | 112 | 160 | 6076 |
| 87 | government | 321 | 746 | 661 | 876 | 408 | 1913 | 91 | 179 | 597 | 54 | 70 | 82 | 5998 |
| 88 | field | 2842 | 571 | 84 | 242 | 1676 | 154 | 18 | 20 | 80 | 15 | 120 | 108 | 5930 |
| 89 | report | 166 | 585 | 89 | 3747 | 259 | 157 | 351 | 33 | 37 | 75 | 235 | 74 | 5808 |
| 90 | interpretation | 138 | 821 | 83 | 3387 | 351 | 131 | 408 | 10 | 70 | 60 | 197 | 121 | 5777 |
| 91 | colleague | 878 | 896 | 300 | 826 | 967 | 578 | 100 | 47 | 496 | 154 | 100 | 274 | 5616 |
| 92 | conclusion | 446 | 511 | 282 | 896 | 657 | 1293 | 302 | 117 | 235 | 268 | 181 | 306 | 5494 |
| 93 | financial assistance | 716 | 622 | 1505 | 604 | 366 | 935 | 48 | 39 | 490 | 65 | 17 | 87 | 5494 |
| 94 | receipt | 101 | 249 | 275 | 1590 | 122 | 377 | 1143 | 3 | 17 | 427 | 515 | 675 | 5494 |
| 95 | second author | 235 | 54 | 29 | 93 | 231 | 689 | 52 | 3203 | 186 | 244 | 305 | 142 | 5463 |
| 96 | partial support | 185 | 368 | 911 | 288 | 753 | 874 | 42 | 377 | 1421 | 42 | 20 | 49 | 5330 |
| 97 | consultant | 26 | 299 | 8 | 4730 | 16 | 8 | 105 | 0 | 3 | 10 | 77 | 8 | 5290 |
| 98 | critical reading | 596 | 2614 | 297 | 1066 | 150 | 155 | 7 | 13 | 353 | 3 | 10 | 11 | 5275 |
| 99 | time | 445 | 381 | 406 | 1262 | 632 | 351 | 522 | 90 | 387 | 179 | 242 | 330 | 5227 |
| 100 | team | 530 | 662 | 205 | 775 | 1747 | 549 | 134 | 29 | 329 | 56 | 100 | 106 | 5222 |
| 101 | referee | 173 | 95 | 103 | 60 | 1235 | 409 | 20 | 2061 | 555 | 151 | 15 | 336 | 5213 |
| 102 | addition | 454 | 636 | 377 | 992 | 650 | 846 | 152 | 93 | 432 | 143 | 117 | 141 | 5033 |
| 103 | useful comment | 731 | 322 | 130 | 150 | 1375 | 378 | 59 | 351 | 781 | 173 | 59 | 493 | 5002 |
| 104 | present study | 485 | 958 | 127 | 2258 | 529 | 273 | 72 | 6 | 156 | 24 | 91 | 19 | 4998 |
| 105 | group | 389 | 858 | 727 | 736 | 640 | 637 | 77 | 120 | 629 | 50 | 55 | 61 | 4979 |
| 106 | hospitality | 246 | 119 | 60 | 47 | 815 | 124 | 12 | 879 | 2433 | 41 | 14 | 182 | 4972 |
| 107 | research work | 425 | 447 | 723 | 428 | 448 | 1808 | 22 | 149 | 399 | 61 | 14 | 33 | 4957 |
| 108 | quality | 606 | 190 | 91 | 180 | 1872 | 1019 | 25 | 353 | 239 | 164 | 45 | 122 | 4906 |
| 109 | authorship | 8 | 124 | 115 | 1521 | 24 | 318 | 1162 | 1 | 2 | 419 | 521 | 677 | 4892 |
| 110 | fieldwork | 2202 | 395 | 9 | 202 | 1548 | 72 | 49 | 1 | 11 | 25 | 60 | 290 | 4864 |
| 111 | student | 820 | 562 | 377 | 733 | 645 | 635 | 185 | 76 | 228 | 249 | 217 | 132 | 4859 |
| 112 | permission | 1604 | 535 | 212 | 354 | 1046 | 421 | 70 | 24 | 106 | 44 | 150 | 194 | 4760 |
| 113 | finding | 391 | 413 | 225 | 681 | 581 | 1035 | 276 | 88 | 125 | 259 | 194 | 286 | 4554 |
| 114 | organization | 223 | 925 | 150 | 2336 | 224 | 185 | 193 | 26 | 65 | 57 | 62 | 83 | 4529 |
| 115 | associate editor | 204 | 56 | 5 | 92 | 454 | 2318 | 5 | 365 | 528 | 226 | 16 | 150 | 4419 |
| 116 | draft | 1209 | 270 | 18 | 302 | 640 | 143 | 178 | 110 | 222 | 322 | 254 | 750 | 4418 |
| 117 | research support | 131 | 278 | 146 | 3081 | 91 | 131 | 77 | 45 | 63 | 103 | 95 | 177 | 4418 |
| 118 | postdoctoral fellowship | 480 | 1316 | 735 | 881 | 241 | 264 | 52 | 123 | 166 | 12 | 91 | 38 | 4399 |
| 119 | patient | 21 | 916 | 20 | 3052 | 18 | 20 | 225 | 5 | 8 | 4 | 99 | 8 | 4396 |
| 120 | valuable discussion | 232 | 598 | 672 | 246 | 473 | 665 | 4 | 124 | 1198 | 10 | 17 | 22 | 4261 |
| 121 | grant agreement | 265 | 848 | 345 | 778 | 648 | 694 | 59 | 96 | 328 | 57 | 35 | 86 | 4239 |
| 122 | institution | 246 | 288 | 68 | 2430 | 417 | 138 | 149 | 83 | 155 | 44 | 45 | 118 | 4181 |
| 123 | information | 718 | 359 | 158 | 578 | 1081 | 504 | 176 | 39 | 217 | 81 | 73 | 172 | 4156 |
| 124 | honorarium | 3 | 261 | 13 | 3575 | 0 | 3 | 82 | 0 | 0 | 3 | 46 | 2 | 3988 |
| 125 | financial interest | 40 | 928 | 164 | 2429 | 41 | 56 | 133 | 1 | 65 | 2 | 54 | 12 | 3925 |
| 126 | recommendation | 524 | 389 | 259 | 249 | 565 | 1009 | 48 | 108 | 196 | 184 | 111 | 178 | 3820 |
| 127 | acknowledges | 79 | 592 | 721 | 91 | 912 | 466 | 4 | 31 | 897 | 2 | 6 | 7 | 3808 |
| 128 | data analysis | 291 | 760 | 118 | 1654 | 319 | 146 | 202 | 4 | 113 | 37 | 138 | 25 | 3807 |
| 129 | science | 351 | 640 | 480 | 665 | 448 | 524 | 70 | 132 | 279 | 49 | 65 | 103 | 3806 |
| 130 | acknowledges support | 42 | 554 | 518 | 76 | 993 | 468 | 0 | 21 | 1050 | 1 | 2 | 6 | 3731 |
| 131 | employee | 153 | 574 | 66 | 2375 | 97 | 127 | 213 | 4 | 38 | 24 | 35 | 20 | 3726 |
| 132 | family | 271 | 820 | 35 | 1474 | 171 | 42 | 212 | 8 | 27 | 59 | 522 | 77 | 3718 |
| 133 | province | 350 | 543 | 637 | 765 | 181 | 713 | 21 | 108 | 349 | 16 | 7 | 15 | 3705 |
| 134 | cooperation | 581 | 402 | 214 | 775 | 639 | 444 | 118 | 16 | 169 | 62 | 107 | 97 | 3624 |
| 135 | conduct | 51 | 365 | 10 | 2680 | 22 | 19 | 308 | 0 | 11 | 19 | 92 | 9 | 3586 |
| 136 | valuable suggestion | 547 | 321 | 262 | 225 | 590 | 565 | 12 | 486 | 315 | 102 | 32 | 110 | 3567 |
| 137 | studentship | 287 | 955 | 545 | 740 | 378 | 224 | 56 | 31 | 207 | 6 | 76 | 42 | 3547 |
| 138 | company | 272 | 422 | 205 | 1670 | 209 | 470 | 53 | 14 | 111 | 35 | 42 | 21 | 3524 |
| 139 | insightful comment | 434 | 261 | 88 | 179 | 920 | 493 | 48 | 200 | 175 | 254 | 89 | 362 | 3503 |
| 140 | interpretation of data | 64 | 442 | 22 | 2414 | 46 | 28 | 288 | 2 | 2 | 34 | 120 | 26 | 3488 |
| 141 | helpful suggestion | 493 | 464 | 227 | 259 | 675 | 340 | 23 | 382 | 210 | 112 | 79 | 218 | 3482 |
| 142 | effort | 435 | 312 | 118 | 729 | 821 | 387 | 129 | 22 | 262 | 46 | 117 | 67 | 3445 |
| 143 | equipment | 436 | 463 | 695 | 491 | 328 | 572 | 21 | 4 | 320 | 5 | 24 | 15 | 3374 |
| 144 | participation | 265 | 554 | 90 | 1198 | 384 | 181 | 199 | 21 | 111 | 72 | 203 | 89 | 3367 |
| 145 | guidance | 507 | 381 | 210 | 463 | 503 | 454 | 134 | 96 | 174 | 143 | 71 | 203 | 3339 |
| 146 | approval | 352 | 394 | 265 | 1521 | 102 | 148 | 184 | 6 | 197 | 13 | 65 | 26 | 3273 |
| 147 | research funding | 137 | 294 | 166 | 2040 | 129 | 198 | 78 | 10 | 51 | 36 | 38 | 68 | 3245 |
| 148 | fee | 9 | 120 | 6 | 2994 | 6 | 8 | 63 | 1 | 0 | 1 | 24 | 0 | 3232 |
| 149 | investigation | 305 | 369 | 383 | 708 | 405 | 682 | 47 | 31 | 189 | 22 | 55 | 25 | 3221 |
| 150 | research fellowship | 297 | 468 | 1096 | 495 | 200 | 309 | 22 | 51 | 227 | 11 | 20 | 16 | 3212 |
| 151 | figure | 398 | 491 | 137 | 423 | 1207 | 159 | 15 | 25 | 204 | 9 | 41 | 75 | 3184 |
| 152 | observation | 110 | 87 | 118 | 26 | 2542 | 151 | 0 | 13 | 77 | 20 | 7 | 17 | 3168 |
| 153 | appreciation | 408 | 252 | 335 | 467 | 359 | 798 | 87 | 66 | 144 | 68 | 67 | 80 | 3131 |
| 154 | excellent technical assistance | 341 | 1115 | 121 | 1473 | 25 | 35 | 1 | 2 | 7 | 0 | 8 | 0 | 3128 |
| 155 | encouragement | 412 | 351 | 391 | 233 | 461 | 423 | 27 | 199 | 351 | 64 | 17 | 152 | 3081 |
| 156 | grant support | 44 | 321 | 46 | 2317 | 30 | 33 | 65 | 6 | 21 | 9 | 31 | 10 | 2933 |
| 157 | code | 154 | 232 | 240 | 243 | 844 | 422 | 11 | 97 | 557 | 36 | 13 | 81 | 2930 |
| 158 | university | 183 | 337 | 445 | 431 | 261 | 522 | 46 | 155 | 330 | 71 | 40 | 109 | 2930 |
| 159 | management | 314 | 336 | 119 | 1415 | 211 | 144 | 152 | 3 | 52 | 23 | 76 | 42 | 2887 |
| 160 | statistical analysis | 597 | 509 | 34 | 1317 | 142 | 48 | 64 | 5 | 13 | 19 | 90 | 29 | 2867 |
| 161 | funding source | 88 | 410 | 32 | 1757 | 72 | 47 | 216 | 2 | 10 | 26 | 151 | 30 | 2841 |
| 162 | other author | 26 | 188 | 13 | 2434 | 16 | 19 | 53 | 12 | 12 | 2 | 57 | 4 | 2836 |
| 163 | software | 199 | 364 | 259 | 325 | 900 | 340 | 17 | 42 | 243 | 18 | 31 | 24 | 2762 |
| 164 | research fund | 191 | 405 | 236 | 816 | 145 | 476 | 77 | 71 | 181 | 59 | 36 | 51 | 2744 |
| 165 | frame | 230 | 296 | 459 | 258 | 312 | 683 | 6 | 70 | 378 | 10 | 7 | 33 | 2742 |
| 166 | form | 242 | 506 | 540 | 377 | 315 | 353 | 33 | 50 | 222 | 16 | 20 | 40 | 2714 |
| 167 | calculation | 25 | 178 | 867 | 59 | 301 | 390 | 4 | 16 | 847 | 4 | 4 | 8 | 2703 |
| 168 | present work | 206 | 298 | 390 | 364 | 224 | 732 | 17 | 96 | 338 | 16 | 14 | 7 | 2702 |
| 169 | investigator | 31 | 887 | 30 | 1503 | 33 | 35 | 76 | 16 | 5 | 10 | 33 | 16 | 2675 |
| 170 | subject matter | 3 | 639 | 91 | 1821 | 4 | 6 | 88 | 4 | 3 | 2 | 0 | 4 | 2665 |
| 171 | scheme | 164 | 292 | 411 | 428 | 248 | 527 | 52 | 58 | 305 | 41 | 38 | 56 | 2620 |
| 172 | specimen | 1639 | 326 | 32 | 109 | 352 | 114 | 0 | 0 | 20 | 0 | 5 | 23 | 2620 |
| 173 | computational resource | 33 | 284 | 770 | 51 | 230 | 496 | 5 | 29 | 702 | 4 | 2 | 5 | 2611 |
| 174 | additional support | 308 | 512 | 163 | 621 | 411 | 202 | 107 | 20 | 142 | 23 | 51 | 46 | 2606 |
| 175 | trial | 204 | 326 | 5 | 1840 | 8 | 25 | 133 | 4 | 1 | 1 | 45 | 3 | 2595 |
| 176 | endorsement | 859 | 264 | 81 | 382 | 481 | 213 | 101 | 9 | 116 | 26 | 30 | 30 | 2592 |
| 177 | entity | 7 | 641 | 93 | 1686 | 7 | 16 | 92 | 0 | 1 | 4 | 6 | 3 | 2556 |
| 178 | image | 314 | 303 | 496 | 209 | 444 | 495 | 0 | 8 | 209 | 4 | 7 | 36 | 2525 |
| 179 | initiative | 210 | 577 | 183 | 588 | 237 | 249 | 94 | 21 | 195 | 34 | 34 | 60 | 2482 |
| 180 | presentation | 86 | 115 | 29 | 214 | 485 | 465 | 34 | 683 | 166 | 98 | 19 | 76 | 2470 |
| 181 | interest | 145 | 364 | 54 | 1175 | 124 | 110 | 112 | 100 | 159 | 21 | 71 | 32 | 2467 |
| 182 | speaker | 21 | 104 | 10 | 2205 | 15 | 8 | 37 | 0 | 5 | 3 | 36 | 2 | 2446 |
| 183 | financial involvement | 0 | 619 | 91 | 1632 | 0 | 2 | 85 | 0 | 0 | 0 | 0 | 0 | 2429 |
| 184 | financial conflict | 0 | 627 | 89 | 1597 | 0 | 4 | 85 | 0 | 0 | 1 | 3 | 0 | 2406 |
| 185 | simulation | 40 | 181 | 228 | 48 | 895 | 368 | 8 | 39 | 555 | 5 | 9 | 13 | 2389 |
| 186 | system | 183 | 305 | 220 | 282 | 549 | 412 | 14 | 32 | 341 | 18 | 21 | 11 | 2388 |
| 187 | generous support | 120 | 328 | 550 | 424 | 190 | 220 | 60 | 64 | 135 | 89 | 38 | 141 | 2359 |
| 188 | crew | 615 | 233 | 24 | 28 | 1351 | 30 | 0 | 0 | 56 | 4 | 8 | 9 | 2358 |
| 189 | researcher | 335 | 345 | 146 | 550 | 206 | 279 | 136 | 24 | 92 | 75 | 57 | 107 | 2352 |
| 190 | third author | 84 | 16 | 8 | 44 | 74 | 362 | 21 | 1385 | 80 | 111 | 116 | 51 | 2352 |
| 191 | partial financial support | 152 | 147 | 525 | 120 | 170 | 535 | 9 | 90 | 534 | 24 | 6 | 34 | 2346 |
| 192 | technology | 197 | 405 | 305 | 513 | 132 | 496 | 20 | 24 | 205 | 21 | 7 | 15 | 2340 |
| 193 | agreement | 179 | 312 | 154 | 522 | 354 | 342 | 42 | 65 | 248 | 28 | 33 | 48 | 2327 |
| 194 | survey | 483 | 132 | 7 | 583 | 436 | 100 | 196 | 9 | 4 | 115 | 55 | 201 | 2321 |
| 195 | corresponding author | 97 | 134 | 54 | 365 | 752 | 607 | 42 | 125 | 60 | 41 | 17 | 26 | 2320 |
| 196 | other relevant affiliation | 0 | 607 | 89 | 1533 | 0 | 2 | 80 | 0 | 0 | 0 | 0 | 0 | 2311 |
| 197 | many thanks | 531 | 192 | 99 | 191 | 535 | 224 | 59 | 46 | 92 | 59 | 76 | 165 | 2269 |
| 198 | feedback | 283 | 272 | 48 | 186 | 350 | 284 | 115 | 31 | 120 | 188 | 137 | 249 | 2263 |
| 199 | funding agency | 115 | 369 | 71 | 756 | 180 | 228 | 189 | 8 | 133 | 59 | 104 | 49 | 2261 |
| 200 | service | 142 | 312 | 214 | 734 | 384 | 176 | 69 | 7 | 132 | 12 | 24 | 28 | 2234 |
| 201 | consortium | 119 | 454 | 156 | 478 | 426 | 253 | 30 | 17 | 218 | 13 | 24 | 31 | 2219 |
| 202 | input | 339 | 299 | 77 | 476 | 371 | 197 | 112 | 18 | 84 | 69 | 58 | 105 | 2205 |
| 203 | sincere thanks | 376 | 142 | 276 | 186 | 298 | 468 | 34 | 122 | 168 | 34 | 9 | 62 | 2175 |
| 204 | research program | 230 | 302 | 213 | 274 | 237 | 485 | 24 | 47 | 222 | 35 | 29 | 53 | 2151 |
| 205 | conference | 63 | 90 | 15 | 279 | 103 | 270 | 79 | 65 | 82 | 313 | 36 | 739 | 2134 |
| 206 | logistical support | 1081 | 189 | 14 | 138 | 536 | 38 | 20 | 0 | 9 | 7 | 49 | 45 | 2126 |
| 207 | visit | 357 | 109 | 84 | 38 | 342 | 195 | 2 | 493 | 418 | 21 | 6 | 48 | 2113 |
| 208 | database | 203 | 219 | 25 | 423 | 945 | 90 | 36 | 13 | 31 | 30 | 12 | 49 | 2076 |
| 209 | thesis | 346 | 266 | 121 | 436 | 320 | 152 | 82 | 135 | 89 | 22 | 46 | 56 | 2071 |
| 210 | course | 267 | 291 | 138 | 272 | 252 | 286 | 41 | 37 | 230 | 66 | 28 | 143 | 2051 |
| 211 | reference | 173 | 257 | 167 | 320 | 266 | 291 | 29 | 210 | 212 | 44 | 29 | 49 | 2047 |
| 212 | product | 470 | 189 | 64 | 519 | 491 | 170 | 32 | 5 | 30 | 19 | 9 | 30 | 2028 |
| 213 | workshop | 302 | 130 | 40 | 152 | 340 | 85 | 45 | 83 | 281 | 153 | 19 | 393 | 2023 |
| 214 | policy | 87 | 425 | 65 | 563 | 204 | 167 | 178 | 14 | 53 | 81 | 80 | 99 | 2016 |
