# Supplementary material for: Beyond funding: Acknowledgement patterns in biomedical, natural and social sciences
Source: PLoS One. 2017 Oct 4;12(10):e0185578. doi: 10.1371/journal.pone.0185578 (PMC5627922; doi:10.1371/journal.pone.0185578)
Supplement: S2 Table — (DOCX) [file pone.0185578.s003.docx]

S2 Table. Quality of representation of the rows (cumulative contribution for each NP)

| **Row** | **NP** | **Axis1** | **Axis2** | **Axis3** | **Axis4** | **Axis5** |
| --- | --- | --- | --- | --- | --- | --- |
| 1 | work | 11.70 | 77.90 | 88.80 | 92.00 | 92.90 |
| 2 | author | 45.40 | 48.70 | 76.80 | 87.80 | 89.10 |
| 3 | grant | 82.20 | 83.30 | 84.10 | 87.50 | 97.10 |
| 4 | study | 56.30 | 78.60 | 92.10 | 97.20 | 98.80 |
| 5 | research | 39.50 | 61.20 | 62.30 | 79.30 | 85.40 |
| 6 | project | 85.20 | 89.70 | 89.80 | 93.20 | 93.30 |
| 7 | financial support | 38.70 | 61.30 | 73.40 | 74.20 | 86.30 |
| 8 | support | 69.90 | 84.80 | 86.10 | 86.50 | 86.90 |
| 9 | manuscript | 33.80 | 34.00 | 83.20 | 94.90 | 97.10 |
| 10 | paper | 45.10 | 68.40 | 73.30 | 83.40 | 83.50 |
| 11 | program | 48.50 | 87.70 | 88.60 | 93.30 | 93.90 |
| 12 | analysis | 69.00 | 77.50 | 96.50 | 96.80 | 98.40 |
| 13 | funding | 4.50 | 5.80 | 12.90 | 61.50 | 73.90 |
| 14 | preparation | 59.10 | 71.20 | 96.70 | 98.50 | 99.60 |
| 15 | assistance | 1.80 | 4.90 | 9.40 | 74.40 | 74.50 |
| 16 | anonymous reviewer | 24.50 | 54.90 | 80.50 | 97.60 | 98.30 |
| 17 | data collection | 59.20 | 69.40 | 96.10 | 97.60 | 99.70 |
| 18 | help | 14.10 | 14.30 | 28.20 | 80.10 | 80.80 |
| 19 | data | 1.20 | 35.20 | 42.10 | 60.80 | 66.30 |
| 20 | decision | 64.60 | 75.60 | 96.60 | 98.40 | 99.40 |
| 21 | thanks | 58.90 | 60.50 | 60.90 | 77.80 | 78.90 |
| 22 | article | 3.20 | 52.00 | 53.00 | 60.90 | 95.40 |
| 23 | funder | 54.80 | 68.10 | 95.70 | 97.60 | 99.30 |
| 24 | role in study design | 50.50 | 66.10 | 96.40 | 98.20 | 99.50 |
| 25 | comment | 20.40 | 56.10 | 91.60 | 94.40 | 94.60 |
| 26 | fellowship | 8.20 | 47.50 | 47.50 | 52.80 | 54.50 |
| 27 | fund | 42.60 | 56.70 | 59.80 | 68.40 | 69.60 |
| 28 | suggestion | 38.60 | 65.20 | 89.50 | 94.60 | 94.70 |
| 29 | contract | 26.80 | 27.30 | 28.30 | 39.70 | 56.60 |
| 30 | number | 79.60 | 79.60 | 89.80 | 90.00 | 91.30 |
| 31 | publication | 1.10 | 1.50 | 21.30 | 21.30 | 21.40 |
| 32 | helpful discussion | 14.60 | 66.50 | 66.60 | 66.60 | 67.50 |
| 33 | research grant | 51.40 | 61.00 | 90.70 | 90.90 | 93.80 |
| 34 | technical assistance | 45.00 | 49.90 | 53.40 | 70.50 | 71.40 |
| 35 | member | 90.50 | 90.80 | 96.20 | 96.30 | 96.50 |
| 36 | contribution | 3.90 | 45.90 | 47.90 | 80.30 | 82.00 |
| 37 | discussion | 21.40 | 31.20 | 38.10 | 38.70 | 43.30 |
| 38 | helpful comment | 17.20 | 59.30 | 80.80 | 82.10 | 90.00 |
| 39 | staff | 1.80 | 31.10 | 34.20 | 71.40 | 73.10 |
| 40 | material | 26.60 | 29.70 | 56.10 | 56.60 | 60.60 |
| 41 | view | 0.10 | 50.40 | 55.50 | 59.10 | 89.70 |
| 42 | conflict of interest | 72.30 | 80.90 | 97.70 | 97.70 | 99.50 |
| 43 | content | 63.20 | 77.90 | 88.90 | 91.10 | 92.50 |
| 44 | scholarship | 0.30 | 2.90 | 3.00 | 31.10 | 31.30 |
| 45 | laboratory | 39.70 | 46.20 | 73.40 | 87.70 | 87.90 |
| 46 | facility | 28.20 | 73.50 | 74.50 | 90.80 | 91.20 |
| 47 | experiment | 13.50 | 41.80 | 42.20 | 61.10 | 62.20 |
| 48 | use | 30.60 | 31.90 | 37.30 | 67.60 | 72.50 |
| 49 | result | 56.50 | 56.50 | 58.80 | 58.80 | 63.40 |
| 50 | first author | 12.10 | 21.50 | 27.20 | 77.60 | 86.90 |
| 51 | award | 17.60 | 40.00 | 46.70 | 50.70 | 51.00 |
| 52 | sponsor | 77.80 | 80.30 | 90.20 | 90.20 | 94.10 |
| 53 | access | 20.60 | 21.90 | 39.50 | 86.20 | 86.30 |
| 54 | framework | 67.70 | 90.60 | 92.60 | 92.90 | 93.10 |
| 55 | review | 2.90 | 3.00 | 27.90 | 28.40 | 30.70 |
| 56 | valuable comment | 44.50 | 67.80 | 90.40 | 93.00 | 94.10 |
| 57 | technical support | 0.00 | 32.90 | 32.90 | 78.70 | 80.30 |
| 58 | anonymous referee | 24.10 | 54.80 | 70.70 | 85.90 | 88.00 |
| 59 | editor | 26.70 | 65.80 | 82.20 | 85.60 | 90.60 |
| 60 | constructive comment | 27.00 | 51.20 | 76.00 | 84.50 | 90.00 |
| 61 | reviewer | 49.20 | 71.50 | 88.20 | 88.30 | 90.00 |
| 62 | education | 42.50 | 83.10 | 90.60 | 90.90 | 90.90 |
| 63 | collection | 42.90 | 74.40 | 88.90 | 92.60 | 94.10 |
| 64 | responsibility | 37.40 | 62.70 | 81.50 | 85.00 | 92.00 |
| 65 | useful discussion | 22.90 | 37.20 | 37.40 | 37.40 | 42.80 |
| 66 | research project | 43.50 | 64.40 | 64.80 | 65.80 | 85.40 |
| 67 | special thanks | 23.50 | 51.30 | 65.40 | 91.50 | 92.00 |
| 68 | measurement | 32.70 | 71.70 | 82.90 | 85.80 | 88.20 |
| 69 | version | 10.10 | 62.10 | 79.40 | 79.60 | 90.40 |
| 70 | recipient | 93.40 | 94.20 | 94.20 | 94.50 | 95.40 |
| 71 | grant sponsor | 63.70 | 69.20 | 69.50 | 71.50 | 71.50 |
| 72 | gratitude | 80.60 | 82.80 | 83.30 | 83.40 | 84.10 |
| 73 | advice | 1.00 | 8.90 | 59.20 | 81.50 | 81.90 |
| 74 | role | 62.80 | 75.10 | 92.50 | 93.00 | 93.00 |
| 75 | writing | 50.40 | 71.60 | 93.90 | 94.70 | 94.80 |
| 76 | participant | 0.60 | 46.70 | 46.80 | 52.50 | 91.40 |
| 77 | official view | 46.90 | 63.60 | 81.50 | 83.60 | 85.80 |
| 78 | fruitful discussion | 30.60 | 59.50 | 60.90 | 60.90 | 63.20 |
| 79 | opinion | 12.50 | 36.10 | 37.30 | 42.20 | 75.20 |
| 80 | study design | 96.70 | 97.40 | 98.40 | 99.10 | 99.10 |
| 81 | design | 46.00 | 60.80 | 87.80 | 88.10 | 88.30 |
| 82 | foundation | 21.70 | 38.30 | 68.70 | 74.40 | 77.40 |
| 83 | sample | 4.60 | 7.80 | 29.90 | 89.30 | 94.90 |
| 84 | collaboration | 37.70 | 47.10 | 47.60 | 63.60 | 65.20 |
| 85 | resource | 24.20 | 61.80 | 70.10 | 72.20 | 72.90 |
| 86 | development | 5.10 | 44.80 | 55.30 | 73.20 | 73.60 |
| 87 | government | 25.20 | 44.90 | 57.40 | 61.10 | 61.80 |
| 88 | field | 4.50 | 17.00 | 36.30 | 70.80 | 75.10 |
| 89 | report | 42.30 | 64.10 | 97.40 | 97.50 | 99.00 |
| 90 | interpretation | 48.70 | 70.60 | 96.90 | 97.00 | 97.40 |
| 91 | colleague | 10.80 | 32.60 | 55.80 | 83.40 | 84.60 |
| 92 | conclusion | 14.80 | 43.10 | 44.20 | 46.40 | 74.40 |
| 93 | financial assistance | 23.00 | 49.50 | 57.40 | 65.80 | 71.50 |
| 94 | receipt | 1.60 | 34.80 | 40.70 | 44.80 | 84.00 |
| 95 | second author | 6.70 | 9.70 | 14.00 | 78.90 | 91.10 |
| 96 | partial support | 48.00 | 67.80 | 69.10 | 72.30 | 77.80 |
| 97 | consultant | 38.50 | 50.20 | 88.10 | 88.10 | 95.00 |
| 98 | critical reading | 53.20 | 73.50 | 93.20 | 94.30 | 94.30 |
| 99 | time | 0.00 | 45.50 | 52.00 | 52.00 | 71.10 |
| 100 | team | 7.90 | 24.40 | 36.70 | 64.70 | 73.50 |
| 101 | referee | 15.60 | 20.40 | 29.50 | 77.60 | 94.10 |
| 102 | addition | 28.40 | 49.40 | 57.60 | 60.60 | 65.60 |
| 103 | useful comment | 40.30 | 58.50 | 79.80 | 79.80 | 80.30 |
| 104 | present study | 56.00 | 67.80 | 78.10 | 84.30 | 96.70 |
| 105 | group | 25.50 | 69.70 | 70.10 | 76.10 | 77.50 |
| 106 | hospitality | 22.50 | 23.90 | 24.90 | 37.40 | 51.30 |
| 107 | research work | 41.60 | 58.50 | 63.60 | 63.60 | 63.90 |
| 108 | quality | 39.90 | 54.70 | 71.30 | 73.10 | 84.50 |
| 109 | authorship | 1.80 | 35.50 | 41.40 | 46.40 | 83.60 |
| 110 | fieldwork | 5.70 | 26.70 | 48.60 | 81.40 | 83.20 |
| 111 | student | 8.90 | 41.10 | 43.30 | 50.60 | 61.20 |
| 112 | permission | 7.20 | 24.20 | 43.10 | 75.90 | 76.30 |
| 113 | finding | 11.80 | 45.80 | 45.90 | 47.40 | 80.00 |
| 114 | organization | 65.40 | 77.10 | 96.30 | 96.30 | 97.20 |
| 115 | associate editor | 40.70 | 40.90 | 41.20 | 53.40 | 53.50 |
| 116 | draft | 7.80 | 52.20 | 63.50 | 64.00 | 81.90 |
| 117 | research support | 30.80 | 50.50 | 87.20 | 87.70 | 89.50 |
| 118 | postdoctoral fellowship | 25.70 | 48.90 | 51.90 | 52.90 | 53.80 |
| 119 | patient | 64.20 | 71.90 | 96.10 | 96.50 | 98.70 |
| 120 | valuable discussion | 28.80 | 66.40 | 67.10 | 67.10 | 69.30 |
| 121 | grant agreement | 3.50 | 11.70 | 18.70 | 23.70 | 29.10 |
| 122 | institution | 27.00 | 56.20 | 85.80 | 85.80 | 93.90 |
| 123 | information | 15.70 | 54.80 | 64.70 | 93.10 | 93.70 |
| 124 | honorarium | 39.60 | 50.40 | 88.00 | 88.10 | 94.90 |
| 125 | financial interest | 68.60 | 71.50 | 94.40 | 94.50 | 96.80 |
| 126 | recommendation | 47.90 | 54.00 | 59.50 | 59.60 | 68.50 |
| 127 | acknowledges | 29.40 | 56.00 | 56.60 | 62.90 | 65.50 |
| 128 | data analysis | 64.50 | 81.30 | 94.20 | 95.70 | 96.70 |
| 129 | science | 41.80 | 52.00 | 52.00 | 52.90 | 53.50 |
| 130 | acknowledges support | 26.70 | 45.30 | 46.40 | 51.40 | 56.50 |
| 131 | employee | 53.40 | 65.10 | 95.60 | 95.60 | 98.00 |
| 132 | family | 32.20 | 49.30 | 51.40 | 51.60 | 54.20 |
| 133 | province | 7.00 | 52.60 | 80.00 | 80.10 | 80.80 |
| 134 | cooperation | 2.30 | 49.00 | 49.30 | 91.90 | 92.30 |
| 135 | conduct | 45.70 | 61.50 | 97.00 | 97.20 | 98.70 |
| 136 | valuable suggestion | 42.10 | 45.70 | 60.80 | 77.50 | 91.20 |
| 137 | studentship | 24.90 | 46.90 | 48.20 | 61.50 | 62.10 |
| 138 | company | 30.50 | 37.70 | 82.60 | 83.80 | 89.90 |
| 139 | insightful comment | 30.90 | 67.60 | 87.90 | 88.10 | 91.70 |
| 140 | interpretation of data | 49.20 | 66.30 | 98.00 | 98.30 | 99.00 |
| 141 | helpful suggestion | 27.60 | 45.40 | 80.10 | 89.70 | 92.50 |
| 142 | effort | 4.90 | 44.00 | 44.60 | 73.70 | 80.90 |
| 143 | equipment | 18.10 | 52.10 | 59.90 | 84.00 | 84.20 |
| 144 | participation | 34.00 | 75.80 | 81.60 | 82.20 | 83.00 |
| 145 | guidance | 20.70 | 69.00 | 77.80 | 79.50 | 93.20 |
| 146 | approval | 36.40 | 45.50 | 87.80 | 89.70 | 90.80 |
| 147 | research funding | 35.00 | 48.50 | 89.90 | 89.90 | 93.20 |
| 148 | fee | 36.10 | 47.60 | 87.00 | 87.10 | 94.70 |
| 149 | investigation | 12.50 | 14.60 | 43.00 | 61.70 | 63.50 |
| 150 | research fellowship | 4.90 | 32.70 | 41.60 | 45.80 | 48.10 |
| 151 | figure | 5.10 | 15.70 | 37.60 | 68.20 | 82.10 |
| 152 | observation | 12.20 | 20.90 | 33.40 | 51.10 | 62.70 |
| 153 | appreciation | 41.30 | 43.10 | 48.40 | 51.30 | 55.80 |
| 154 | excellent technical assistance | 84.50 | 84.50 | 85.30 | 86.50 | 89.40 |
| 155 | encouragement | 72.30 | 72.30 | 83.10 | 83.70 | 84.10 |
| 156 | grant support | 44.30 | 54.00 | 88.80 | 88.80 | 95.00 |
| 157 | code | 46.90 | 47.00 | 50.00 | 53.70 | 64.70 |
| 158 | university | 58.20 | 66.70 | 74.00 | 90.50 | 93.30 |
| 159 | management | 39.70 | 66.20 | 93.80 | 96.70 | 98.50 |
| 160 | statistical analysis | 43.10 | 63.40 | 67.40 | 75.20 | 79.30 |
| 161 | funding source | 51.00 | 70.00 | 97.30 | 97.60 | 97.70 |
| 162 | other author | 39.10 | 50.70 | 88.10 | 88.20 | 95.30 |
| 163 | software | 19.30 | 20.10 | 29.50 | 52.20 | 66.70 |
| 164 | research fund | 9.80 | 10.30 | 74.50 | 81.00 | 81.30 |
| 165 | frame | 56.40 | 91.30 | 95.80 | 97.00 | 97.20 |
| 166 | form | 11.50 | 53.90 | 54.30 | 65.00 | 66.20 |
| 167 | calculation | 30.80 | 68.40 | 75.50 | 76.50 | 76.50 |
| 168 | present work | 40.70 | 74.80 | 87.00 | 87.50 | 88.60 |
| 169 | investigator | 86.30 | 87.60 | 94.70 | 95.30 | 96.70 |
| 170 | subject matter | 66.30 | 69.40 | 91.60 | 91.70 | 94.80 |
| 171 | scheme | 45.90 | 68.60 | 95.70 | 95.70 | 98.90 |
| 172 | specimen | 1.30 | 6.30 | 18.20 | 44.00 | 45.90 |
| 173 | computational resource | 30.70 | 79.70 | 85.80 | 86.10 | 86.50 |
| 174 | additional support | 21.10 | 42.30 | 45.10 | 83.40 | 85.40 |
| 175 | trial | 50.10 | 64.30 | 94.10 | 94.20 | 98.40 |
| 176 | endorsement | 2.20 | 18.50 | 25.80 | 61.80 | 64.80 |
| 177 | entity | 69.30 | 72.00 | 93.00 | 93.10 | 95.50 |
| 178 | image | 43.90 | 60.20 | 60.20 | 88.70 | 88.70 |
| 179 | initiative | 62.90 | 63.00 | 63.00 | 70.50 | 76.70 |
| 180 | presentation | 16.70 | 22.40 | 28.30 | 79.80 | 96.40 |
| 181 | interest | 43.50 | 60.40 | 85.00 | 88.90 | 96.10 |
| 182 | speaker | 36.30 | 48.10 | 87.00 | 87.00 | 94.50 |
| 183 | financial involvement | 69.10 | 71.50 | 92.30 | 92.50 | 95.10 |
| 184 | financial conflict | 70.50 | 72.70 | 92.70 | 92.90 | 95.30 |
| 185 | simulation | 36.90 | 38.90 | 41.60 | 48.90 | 59.60 |
| 186 | system | 38.00 | 42.50 | 44.00 | 60.90 | 74.90 |
| 187 | generous support | 6.50 | 7.20 | 13.30 | 14.10 | 44.40 |
| 188 | crew | 9.00 | 20.80 | 46.20 | 81.00 | 94.30 |
| 189 | researcher | 3.00 | 51.10 | 52.30 | 55.80 | 80.30 |
| 190 | third author | 6.90 | 9.50 | 13.40 | 79.40 | 91.60 |
| 191 | partial financial support | 51.90 | 85.80 | 93.60 | 94.00 | 94.00 |
| 192 | technology | 0.70 | 46.10 | 74.80 | 76.80 | 76.90 |
| 193 | agreement | 13.70 | 18.00 | 24.10 | 27.40 | 59.20 |
| 194 | survey | 0.00 | 77.80 | 78.60 | 85.70 | 92.20 |
| 195 | corresponding author | 21.60 | 33.00 | 34.70 | 35.10 | 48.80 |
| 196 | other relevant affiliation | 70.50 | 72.60 | 92.40 | 92.60 | 95.10 |
| 197 | many thanks | 18.80 | 57.10 | 77.00 | 93.40 | 94.20 |
| 198 | feedback | 8.40 | 47.60 | 56.60 | 56.80 | 91.40 |
| 199 | funding agency | 27.60 | 50.90 | 67.30 | 67.60 | 74.10 |
| 200 | service | 12.80 | 22.90 | 41.30 | 63.20 | 73.70 |
| 201 | consortium | 0.90 | 1.80 | 4.30 | 26.60 | 42.50 |
| 202 | input | 0.10 | 71.40 | 75.30 | 91.00 | 94.90 |
| 203 | sincere thanks | 72.20 | 72.30 | 73.00 | 73.70 | 74.70 |
| 204 | research program | 58.10 | 69.60 | 69.80 | 71.70 | 72.60 |
| 205 | conference | 4.50 | 28.90 | 30.00 | 36.90 | 74.30 |
| 206 | logistical support | 2.80 | 17.20 | 33.00 | 65.80 | 69.00 |
| 207 | visit | 26.30 | 26.90 | 36.40 | 61.30 | 86.70 |
| 208 | database | 2.00 | 26.90 | 37.70 | 60.40 | 73.40 |
| 209 | thesis | 0.60 | 48.10 | 55.40 | 55.50 | 69.00 |
| 210 | course | 28.90 | 39.00 | 46.40 | 48.10 | 64.30 |
| 211 | reference | 29.90 | 31.10 | 34.50 | 76.00 | 97.20 |
| 212 | product | 0.10 | 31.60 | 34.40 | 79.00 | 93.00 |
| 213 | workshop | 15.30 | 42.20 | 50.60 | 51.30 | 65.70 |
| 214 | policy | 23.90 | 56.00 | 56.60 | 57.90 | 82.00 |
