# Supplementary material for: Beyond funding: Acknowledgement patterns in biomedical, natural and social sciences
Source: PLoS One. 2017 Oct 4;12(10):e0185578. doi: 10.1371/journal.pone.0185578 (PMC5627922; doi:10.1371/journal.pone.0185578)
Supplement: S3 Table — (DOCX) [file pone.0185578.s004.docx]

S3 Table. Quality of representation of the columns (cumulative contribution for each discipline)

| **Column** | **Discipline** | **Axis1** | **Axis2** | **Axis3** | **Axis4** | **Axis5** |
| --- | --- | --- | --- | --- | --- | --- |
| 1 | Biology | 8.20 | 21.20 | 33.10 | 61.60 | 62.60 |
| 2 | Biomedical Research | 57.80 | 74.40 | 98.80 | 99.10 | 99.90 |
| 3 | Chemistry | 26.80 | 63.50 | 77.80 | 79.60 | 83.60 |
| 4 | Clinical Medicine | 55.80 | 65.80 | 96.50 | 96.50 | 99.30 |
| 5 | Earth and Space | 30.40 | 48.00 | 65.40 | 81.10 | 88.30 |
| 6 | Engineering and Technology | 58.10 | 68.40 | 76.80 | 78.50 | 79.70 |
| 7 | Health | 10.30 | 41.20 | 50.80 | 52.50 | 72.20 |
| 8 | Mathematics | 12.90 | 15.30 | 21.80 | 85.00 | 96.10 |
| 9 | Physics | 39.40 | 65.40 | 69.20 | 69.50 | 71.30 |
| 10 | Professional Fields | 16.30 | 58.50 | 61.10 | 70.90 | 94.60 |
| 11 | Psychology | 2.50 | 45.40 | 45.50 | 50.20 | 61.20 |
| 12 | Social Sciences | 14.90 | 55.10 | 59.90 | 63.50 | 90.10 |
